# Autologous chyle fat grafting for the treatment of hypertrophic scars and scar-related conditions

**DOI:** 10.1186/s13287-018-0782-8

**Published:** 2018-03-09

**Authors:** Xiao Xu, Linying Lai, Xuyi Zhang, Jinhong Chen, Junnan Chen, Fei Wang, Jingchen Zheng, Minliang Chen

**Affiliations:** 1 Department of Plastic and Reconstructive Surgery, The General Hospital of Chinese People’s Armed Police Forces, No. 69 Yongding Road, Haidian District, Beijing, 100039 China; 2 Department of Burn and Plastic Surgery, the First Affiliated Hospital of Chinese PLA General Hospital, No. 51 Fucheng Road, Haidian District, Beijing, 100038 China; 3 Department of Medical Administration, The General Hospital of Chinese People’s Armed Police Forces, No. 69 Yongding Road, Haidian District, Beijing, 100039 China; 4 Institute of Rescue Medicine, The General Hospital of Chinese People’s Armed Police Forces, No. 69 Yongding Road, Haidian District, Beijing, 100039 China

**Keywords:** Chyle fat, Fat transplantation, Fibroblasts, Collagen, Scar

## Abstract

**Background:**

Scarring is the product of natural restoration, yet its treatment remains challenging. Both collagen and fibroblasts are abnormally abundant in scars, leading to scar hyperplasia or contracture. Several clinical studies have reported that wrinkles at the recipient site are reduced, pores are narrowed, pigmentation is decreased, and skin is softened after autologous fat transplantation. In this study, we investigated the ability of autologous chyle fat injection to normalize the fibroblasts and collagen of scar tissue in 80 adult patients with hypertrophic scars resulting from severe burns received more than 1 year previously.

**Methods:**

The patients underwent autologous chyle fat injection, and scar samples were collected at different time points. Differences in the number of adipocytes before and after chylosis were assessed by cell culture, and changes in the structural organization of the scars were detected via histologic and immunohistochemical analyses.

**Results:**

After preparation, the chyle fat contained few autologous adipocytes and large amounts of extracellular matrix. Following the injection of chyle fat, the thickness, color, and elasticity of hypertrophic scar tissue tended toward normalization, and patient satisfaction increased. The three adipose tissue donor sites used for the preparation of chyle fat were the abdomen, buttocks, and inner thigh, of which the inner thigh yielded the best therapeutic outcomes. The density and quantity of fibroblasts in the scars decreased following the injection of chyle fat, and the arrangement, quantity, and shape of type III collagen fibers tended toward normalization. After three treatments, the results of immunohistochemical staining showed that type III collagen was significantly less abundant than before treatment.

**Conclusions:**

Autologous chyle fat transplantation has a good therapeutic effect on hypertrophic scar tissue. The injection of chyle fat into hypertrophic scar tissue reduced the density and quantity of fibroblasts and prompted the arrangement, quantity, and shape of type III collagen to normalize.

## Background

Hypertrophic scars (HSs) can occur after dermal tissue damage as a consequence of abnormal deposition and remodeling of components of the extracellular matrix (ECM), especially collagen [1]. HS tissue is usually raised and inflexible, exhibiting itching, pain, and redness as a result of overabundant wound matrix, and can cause significant cosmetic and functional problems for patients [2]. Currently, there are many different therapeutic options for the treatment of HSs, including excision, intralesional corticosteroid injection, compression, laser removal, and interferon injection. However, none of these treatments has been confirmed to effectively eliminate excessive scar tissue formation and regenerate healthy dermal tissue [3, 4]. Hence, the treatment of HS tissue remains a challenge for clinicians.

To date, there is no gold-standard therapy for scar treatment. Following the application of autologous fat and adipose-derived stem cell (ADSC) transplantation in regenerative medicine, many researchers are turning to fat and ADSC injection techniques for scar treatment [5–8]. Basic and clinical studies have shown that autologous fat particle transplantation stimulates the regeneration of the dermis and hypodermis and improves the elasticity and extension of scar tissue [9]. Autologous fat particles have been demonstrated to help improve the skin texture, dermal thickness, blood vessel regeneration, and microcirculation of defective areas, indicating their potential for scar treatment [10]. Other studies have shown that ADSCs function in improving skin texture after autologous fat particle injection. Yun et al. [11] found that ADSCs help to inhibit the proliferation of scar tissue by reducing the activity of myofibroblasts and mast cells, preventing transforming growth factor-β1 (TGF-β1) from stimulating fibroblasts (Fbs), and promoting scar collagenous tissue shaping via matrix metalloproteinase-1 expression. These actions have important effects on the formation and reshaping of scars.

In-vitro and animal experiments have shown that coculture of ADSCs and HS-sourced Fbs reduces the proliferation ability of HS-sourced Fbs, decreases the expression of TGF-β1, phosphorylated Smad2/3, and type I/III collagen in the TGF-β1/Smad pathway, blocks the interaction between TGF-β1 and Smad3 and subsequent translocation of Smad3 to the nucleus, and reverses the TGF-β1 and Smad3 signal transduction pathway through crosstalk with other transduction pathways, thus preventing ECM deposition, tissue fibrosis, and pathologic scar formation [12, 13]. The preventive effect of ADSCs on scar tissue is not limited to antifibrotic activity: activated ADSCs also increase the expression of prostaglandin-E2 and epoxidase-2 and decrease the proliferation abilities of cluster of differentiation CD4^+^ and CD8^+^ T lymphocytes, thus avoiding the effects on Fbs of profibrogenic cytokines released by T cells and macrophages, as well as the formation of subsequent scarring [14]. Recent studies have indicated that expression of the tumor suppressor gene p53 in ADSCs is closely connected with the improvement of HSs [15, 16].

Domestic and international reports on the use of autologous fat transplantation in scar treatment are limited to animal experiments, and the treatment of human hypertrophic and pitting scars remains at the fat particle transplantation stage. It is difficult to inject fat particles into dense scars, which is a major obstacle to the promotion of clinical applications of this treatment. Systematic reports involving clinical studies on the protocols for and outcomes of autologous fat transplantation and its effects on Fb vitality, collagen synthesis, and relative cytokine levels in scar tissue are scarce. In this study, we attempted to use a transverter to turn autologous fat into chyle fat to aid local fat injection.

## Methods

Over the last 3 years, we used the fat injection technique to treat 80 patients with postsurgical HSs resulting from severe burns received more than 1 year previously. The procedure was performed under local anesthesia. The donor site for the fat graft was infiltrated with a solution consisting of 500 ml of Ringer’s solution, 15 ml of 5% xylocaine, and 1.3 mg of adrenaline (1/100,000). The area of the scar destined for fat injection was marked in the standing position before surgery. The patient was placed in the supine position, and an iodophor was used to sterilize the treatment area. The patient was covered with a sterilized sheet. The marks made preoperatively were identified. The ethical committee of the First Affiliated Hospital of Chinese PLA General Hospital was approved this study, all patients consented to participate in the research and approved the ethics, and written informed consent was obtained from participants.

### Preparation of chyle fat

Strict asepsis was observed. According to the preoperative markers of liposuction range, a cut approximately 0.5 cm long was made in the direction of the dermatoglyph, and then infiltration solution was injected into the subcutaneous fat layer of the marked area. Fat harvesting and collection were accomplished with a standard liposuction setup using the 20-ml syringe method with a pumpback of 10 ml (the negative pressure was about −60 kPa). The donor sites were the hips, buttocks, thighs, and abdomen. The wet technique was employed to obtain fat with a minimal amount of blood, and Coleman’s technique was used to prepare the fat implant [6]. The harvested fat was filtered, cleaned, purified with Ringer’s solution, depleted of thick fibrous tissue, and pushed back and forth 30 times through an 0.8-mm nanometer transverter to ensure that predominantly chyle fat was kept for injection. The subcutaneous part of the infiltration solution was then extruded, the incision was sutured with 6-0 nylon thread, and a sterile gauze bandage was applied.

### Lipofilling

The infiltration solution was injected under and into a small area on either side of the scar. After a waiting period of 15 min, a 0.5-cm incision was made at a concealed site at one end of the scar. The prepared chyle fat was then directly injected into the scar through a 2-ml injector while simultaneously withdrawing the 21-G sharp needles out of the incision.

After checking that there was no significant bleeding or local obstacles to blood supply, the incision was sutured with 6-0 nylon thread. Subsequently, a dressing was applied to compress the area around the treated area while avoiding pressing on the scar. Each patient was treated three times, with a treatment interval of 3 months.

### Fat culture

The harvested fat particle and chyle fat were digested in trypsin, centrifuged, and precipitated, and the supernatant was discarded. A tissue block approximately 1 mm^3^ in size was removed from the sediment and cultured in culture medium containing calf serum.

### Curative effect

Using the same focal length and light conditions, the same doctor captured images of each patient at subsequent visits, and recorded the thickness, texture, color, and roughness of their scar. Two dermatologists observed and recorded the clinical situation of the fat injected scar area and patient satisfaction, and harvested scar tissue for pathologic examination. They categorized the curative effect into three levels after 2, 4, and 6 months and 1 year as follows:

- Level 1 (obvious): the area of the scar had shrunk by more than 65%, its texture had become softer, its elasticity had increased, and its color was more like that of normal skin. The scar had a minor effect on the patient’s appearance, the patient was satisfied, and there was no recurrence after 1 year.
- Level 2 (effective): the area of the scar had shrunk by more than 30% but less than 65%, it had become thinner and softer, and it exhibited an obvious pigmentation change. There was no recurrence after 1 year; however, the patient was not completely satisfied with the treatment.
- Level 3 (noneffective): the area of the scar area had shrunk by less than 30%, and there were no changes or only minor changes to its texture and color. The scarring recurred after 1 year, and the patient was unsatisfied with the treatment.

### In-vivo analyses

#### Histologic analysis

Samples were obtained from the scars before each treatment for histologic analysis. They were bisected and immediately fixed in 10% formalin. Subsequently, the scars were embedded in paraffin and cut into sections. The sections were stained with hematoxylin and eosin, examined by microscope (Nikon Eclipse E400; Nikon Inc., Tokyo, Japan), and measured using a digital image analysis system (NIS-Elements Basic Research; Nikon Instech Co., Ltd, Tokyo, Japan). The measurements were performed twice by a blinded examiner, and average values were used. Further evaluation of the collagen fiber arrangement was performed via Masson trichrome staining.

### Statistical analysis

Scars were treated and harvested in a matched fashion with their internal controls. GraphPad Prism 6 (GraphPad Software, Inc., La Jolla, CA, USA) was used to analyze the data. Student’s *t* test was used to compare the density of type III collagen in treated scars and their internal controls. The Kruskal–Wallis (rank-sum) test was used to compare the curative effects of different donor sites. Statistical significance was set at *P* < 0.05.

## Results

### In-vivo analyses

**Fig. 1:**
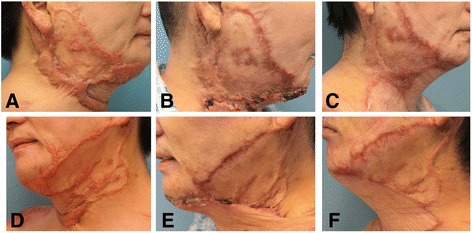
Clinical observation of the hypertrophic scars after chyle fat grafting. **a**, **d** Preoperative view of a 47-year-old patient with faciocervical scars that were hypertrophic. **b**, **e** Postoperative view of the same patient after one chyle fat graft. **c**, **f** Postoperative view of the same patient at a follow-up assessment 1 year after three chyle fat grafts

Our experiences during 3 years of follow-up were as follows. Good results were achieved in all 80 cases, in whom the HS became as even as the adjacent skin. Of these 80 cases after three chyle fat transplantation treatments, the outcomes of 67 were categorized as obvious, eight as effective, and five as noneffective. In addition, the patients reported satisfaction with the results. Figure 1 shows the preoperative and postoperative photographs of a representative patient. In two patients, there was partial recurrence; that is, part of the scar readhered to the underlying fascia. Both of these patients were reoperated on 6 months after the three treatments using the same technique under local anesthesia. The results were good in both of these patients. There were no incidences of infection, irregularity, or seroma at the donor site.

### In-vitro analyses

#### Histologic changes in adipocytes

**Fig. 2:**
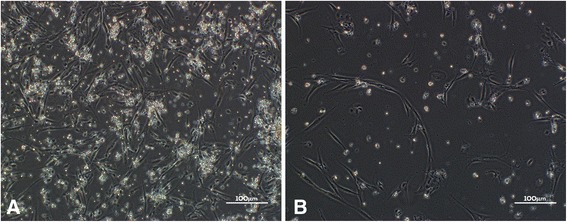
**a** Adipocytes cultured from unchylosis fat. **b** Adipocytes cultured from chyle fat. A great number of strip-shaped adipocytes and fewer lipid droplets were cultured from unchylosis fat. Visibly fewer adipocytes were cultured from chyle fat, and a large amount of cell debris piled up

After culture in Petri dishes under appropriate conditions for 5 days, unprepared and prepared fat samples were examined by optical microscopy. Cultured fat particles exhibited a large number of funicular adipocytes and a small number of lipid droplets (Fig. 2a). Conversely, cultured chyle fat exhibited few funicular adipocytes, but a large amount of accumulated cell debris (Fig. 2b).

### Comparison of the curative effects of fat harvested from different areas

**Fig. 3:**
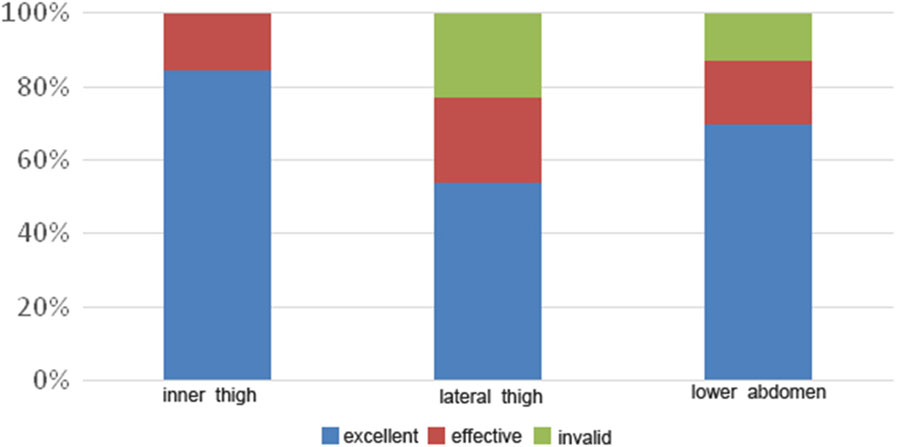
Comparison of curative effects of a range of donor sites. Compared with the lateral thigh and lower abdomen, the inner thigh demonstrated the best curative effect

The total effective rates of chyle fat harvested from different donor sites were as follows: lateral thighs, 100% (*n* = 38); inner thighs, 76% (*n* = 26); and abdomen, 56.88% (*n* = 16). Statistical analysis of the curative effects of different donor sites using the Kruskal–Wallis rank-sum test with a significance level of *P* < 0.05 and according to an inspection level of α = 0.05 identified significant differences between the curative effects of fat harvested from the different donor sites. Fat collected from the inner thighs had a better curative effect than that harvested from the lateral thighs and abdomen, and fat obtained from the lateral thigh achieved superior results to that harvested from the abdomen. Thus, chyle fat transplantation from the inner thigh can be considered to have the best curative effect (Fig. 3).

### Hematoxylin and eosin staining

**Fig. 4:**
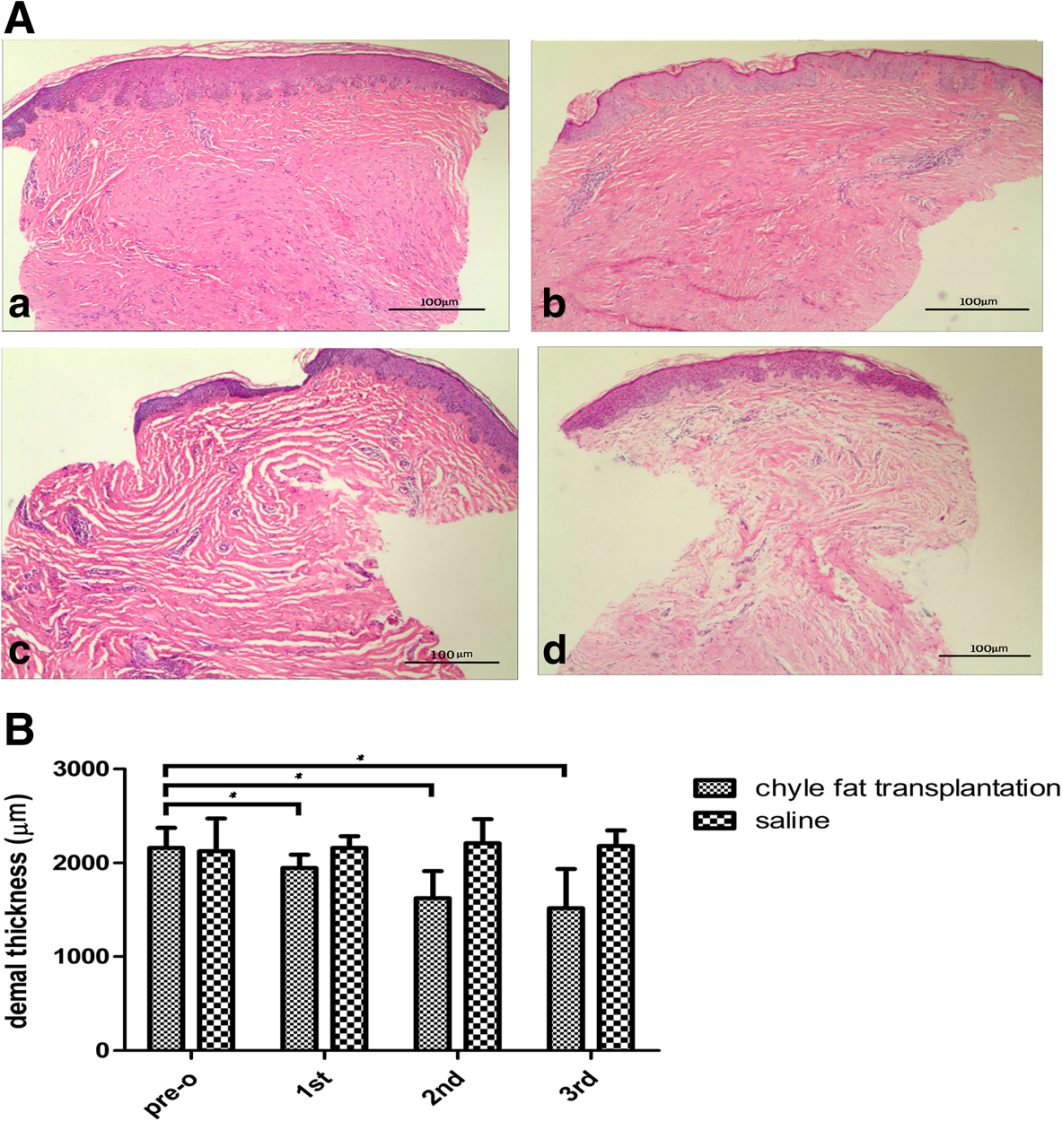
Histopathologic evaluation of a hypertrophic scar. **A** Compared with the preoperative scar (a), after three treatments the dermis was thinner than before (b–d), density and quantity of fibroblasts were decreased, and density of blood vessels was reduced. Each patient was treated three times, with interval between treatments of 3 months. Cells stained with hematoxylin and eosin (magnification × 100). **B** Chyle fat transplantation treatment gradually improved the dermal thickness of hypertrophic scars. *pre-o chyle fat transplantation group compared with 1st, 2nd and 3rd chyle fat transplantation group , *P* < 0.05

Before treatment, the structure of HS tissue was as follows: the epidermis was thinner; the spinous layer was missing; the dermis was thicker; Fbs were enlarged; collagen fibers were thicker, and exhibited disordered alignment in multipolar, swirled, and nodular structures; and blood vessels were more numerous. After three treatments, the thickness of the dermis had decreased, the number and density of Fbs were reduced, collagen fibers in the dermis were more neatly arranged and deeper collagen fiber bundles had become thinner, and the density of blood vessels had declined (Fig. 4).

### Masson staining

**Fig. 5:**
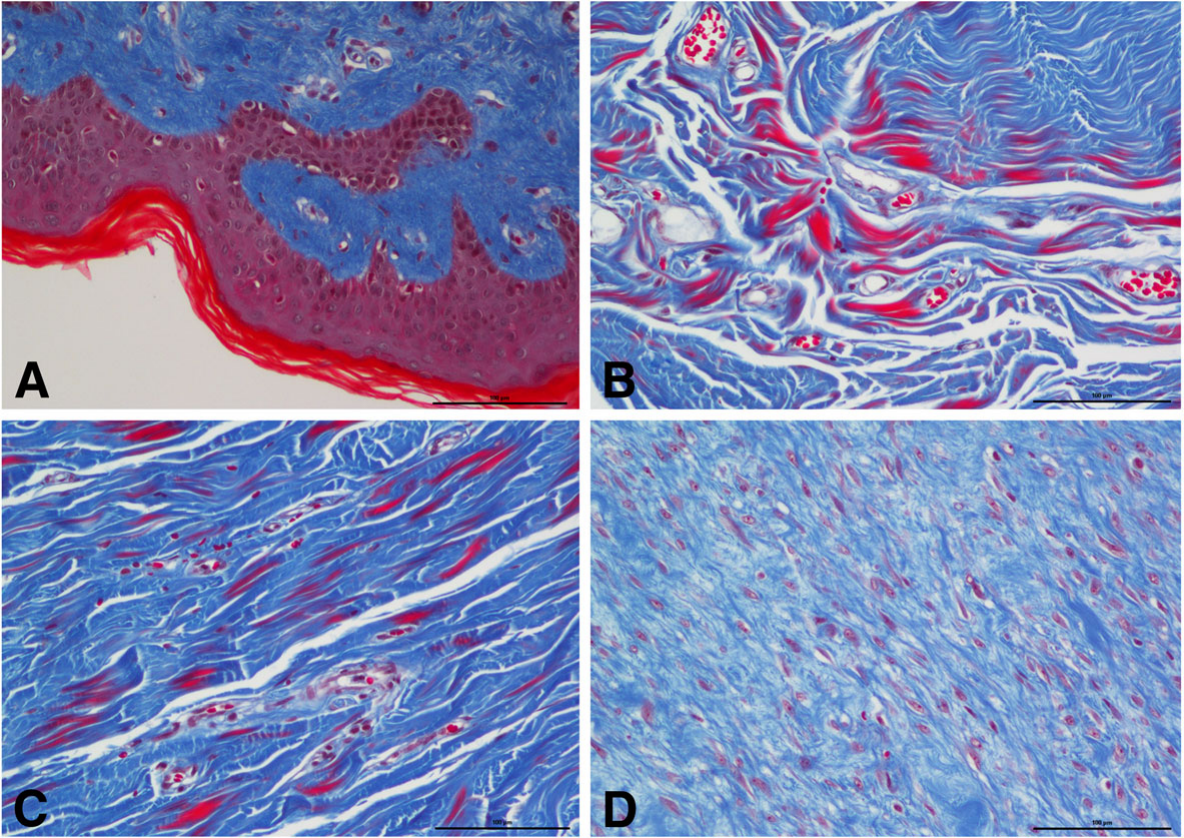
Masson trichrome staining for evaluation of collagen fiber organization. Collagen fibers were dense and disorderly in the untreated scars (**a**). After three chyle fat treatments, collagen fibers gradually became regularly arranged (**b**–**d**). Interval between treatments was 3 months (magnification × 200)

Proliferation of collagen fibrosis in HSs was abundant before treatment. Numerous deep blue-stained collagen fibers were visible, collagen bundles had become thicker and adopted an abnormal shape, and collagen fiber alignment was crowded and disordered. After treatment, collagen fibers in the treated group had dramatically reduced in number, the blue staining had faded, the bundles had become thinner, and the alignment had loosened (Fig. 5).

### Immunohistochemistry

**Fig. 6:**
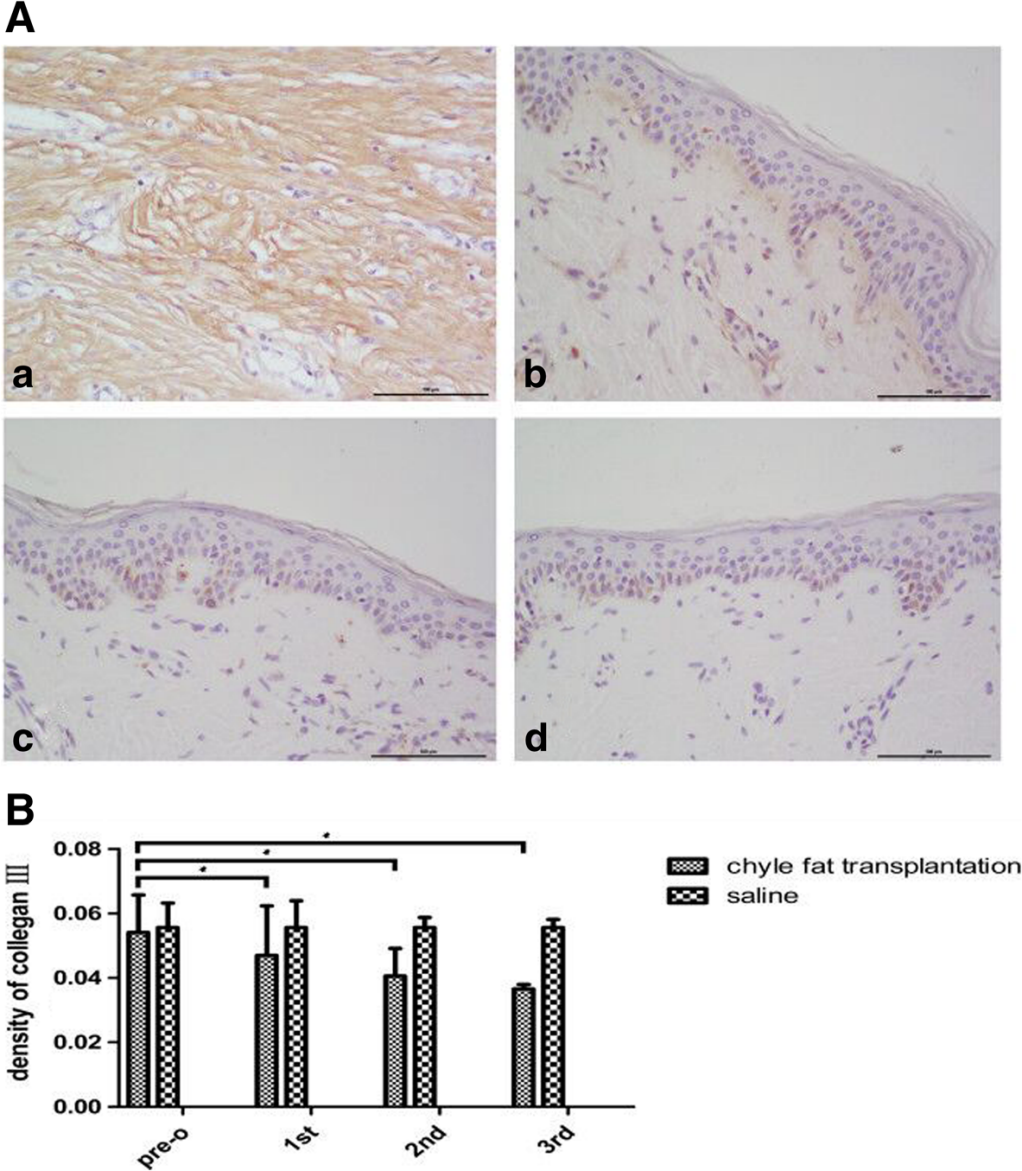
**A** Compared with pre treatment (a), collagen fiber III in hypertrophic scar was thinner ,neat and orderly after each chyle fat grafting (b-d). **B** The density of collagen III in HSs was decreased gradually after each chyle fat grafting. *P < 0.05 compared with pretreatment in the chyle fat group

The results of immunohistochemical analyses showed that, compared with the saline-injected group, the density of type III collagen in HSs decreased significantly following chyle fat transplantation treatment (Fig. 6).

## Discussion

In 2013, Klinger et al. [17] proved that fat particle transplantation can be used to cure HSs. After treatment, the skin regained the softness, elasticity, color, and thickness of normal skin; moreover, histologic analyses identified collagen deposition, blood vessel proliferation, and thickening of the dermis, indicating that scar tissue retains the ability to become normal skin. Also in 2013, Bruno et al. [9] found that fat particle transplantation stimulates the regeneration of elastic fibers under scars, causing disordered collagenous fibers to regain normal alignment and compactness. In recent years, many studies have endeavored to elucidate the underlying mechanism of this repair: because fat tissue is rich in adipocytes, ADSCs, preadipocytes, macrophages, and endotheliocytes, researchers have asserted that these cells not only provide physical support in the injected area, but also secrete cytokines, which are closely connected with regeneration and metabolism. These cytokines may stimulate the regeneration of Fbs and synthesis of collagenous fibers in the recipient area, thicken the dermis, stimulate endotheliocyte proliferation in blood vessels, and hasten the resumption of blood circulation, providing oxygen and nutrition and improving scar texture [18–21].

Compared with the large number of fat cells cultivated from unchyled fat, few mature fat cells were cultivated from chyle fat in this study. Mature fat cells account for only one-sixth of all fat cells, but they are in charge of lipid metabolism, the main function of fat cells. It is established that, as terminally differentiated cells, mature fat cells cannot be subcultured. However, after culturing mature fat cells in vitro, Zuk et al. [22] found that they can jettison their lipid and become Fb shaped, which is more tolerant of an anaerobic environment, and under certain conditions can differentiate into fat, cartilage, and bone cells. We believe that the effects of autologous chyle fat transplantation, either during facial rejuvenation or wound repair treatment, are not caused by the fat cells themselves: preadipocytes play a more important role. However, other researchers propose that the anti-scarring effect of fat cells is closely connected with ADSCs [20].

Discovered by Zuk et al. [23] in 2010, ADSCs are a kind of mesenchymal stem cell, but are more abundant and more readily acquired, separated, and cultured. They are now widely used as seed cells in tissue engineering, as well as in wound healing, whitening, antiaging, and antifibrosis studies [24–28]. ADSCs have the ability to differentiate into several different fat cells. ADSCs are also able to secrete growth factors, such as growth factors that stimulate the formation of blood vessels, which have antiapoptotic, antioxidant, immunoregulatory, and anti-inflammatory characteristics. They can also affect the metabolism of the ECM through Fbs in the dermis. As stem cells, they can give rise to different cell pedigrees in different environments, such as endothelial cells, which control the vascular bed, thickness of collagen, and formation of granulation tissue [21]. Zhang et al. [28] injected human adipose-derived stem cell-conditioned medium (ADSC-CM) into scars on rabbit ears, and successfully decreased the scar proliferation index, amount of type I collagen, and expression of α-smooth muscle actin. Other researchers have studied the composition of ADSC-CM, and found abundant antifibrotic cell factors [29] such as hepatocyte growth factor and interleukin-10.

Chyle fat transplantation decreases the density of type III collagen in HSs; when collagenous fibers are in good order and their shapes are regular, it has an undoubted beneficial effect on HS tissue. Chyle fat is easier to inject than fat particles, so the treatment efficiency is also improved. Because of these advantages, the clinical applications of the technique are worth promoting. Its mechanism of action involves alteration of the ECM in ADSCs; however, whether this is achieved by perturbogens or protein expression requires further investigation.

## Conclusion

In this study, we investigated the antiscarring effect of autologous chyle fat injection in 80 patients with HSs. We speculated that chyle fat would suppress the formation of HSs through more than the secretion of antifibrotic cytokines by ADSCs when injected locally in vivo. This is a preliminary study that, to our knowledge, has not been confirmed by others. Although further investigation of autologous chyle fat is necessary, the technique demonstrates immense potential for clinical application as an antiscarring agent in the field of HS prevention and treatment.

Our preliminary results suggest that autologous chyle fat lipofilling can improve scar quality. We believe that this improvement depends on tissue regeneration promoted by ADSCs. The morbidity related to the procedure is minimal, similar to that for limited liposuction, with acceptable safety. Our results are encouraging and suggest that further research is warranted to assess adipose cell properties, ECM composition, and the essential requisites for routine clinical application.

## Abbreviations

ADSC: Adipose-derived stem cell
ADSC-CM: Adipose-derived stem cell-conditioned medium
CD: Cluster of differentiation
ECM: Extracellular matrix
Fb: Fibroblast
HS: Hypertrophic scar
TGF-β1: Transforming growth factor-β1
